# Efficacy and safety of electroacupuncture plus warm needling therapy for heel pain: study protocol for a randomized controlled trial

**DOI:** 10.1186/s13063-019-3572-4

**Published:** 2019-08-07

**Authors:** Lai Fun Ho, Yuanqi Guo, Jessica Yuet-Ling Ching, Kam Leung Chan, Ping Him Tsang, Man Hin Wong, Liyi Chen, Bacon Fung-Leung Ng, Zhi-Xiu Lin

**Affiliations:** 1 Chinese Medicine Services, Pok Oi Hospital, Hong Kong SAR, China; 2 School of Chinese Medicine, Faculty of Medicine, The Chinese University of Hong Kong, Hong Kong SAR, China; 3 Hong Kong Institute of Integrative Medicine, Faculty of Medicine, The Chinese University of Hong Kong, Hong Kong SAR, China; 4 Chinese Medicine Department, Hospital Authority, Hong Kong SAR, China

**Keywords:** Electroacupuncture, Warm needling, Acupuncture, Moxibustion, Heel pain, Randomized controlled trial

## Abstract

**Background:**

Heel pain is a common foot disorder that causes pain and functional limitations. The prevalence of disabling foot pain will increase as the population ages. Previous studies have reported the positive therapeutic effects of electroacupuncture, warm needling, or the combination of both for heel pain but with limitations in the study methodologies. The current study is a rigorously designed randomized controlled trial that aims to evaluate the clinical efficacy and safety of electroacupuncture plus warm needling therapy in patients with heel pain.

**Methods/design:**

The study protocol describes a prospective, open-label, parallel-group, randomized controlled trial to be conducted in Hong Kong. Eighty patients aged 50–80 years who have reported heel pain and first-step pain equal to or exceeding 50 mm on the 100-mm visual analog scale (VAS) will be recruited. They will be randomly assigned (1:1 ratio) to the electroacupuncture plus warm needling therapy (i.e., treatment) group or the waitlist (i.e., control) group. The treatment group will undergo six treatment sessions in 4 weeks. The control group will receive no treatment during the study period. The primary outcome measure is a mean change in the first-step pain VAS score from the baseline to week 4. Secondary outcome measures include a mean change in first-step pain VAS score from the baseline to week 2, a mean change in Foot Function Index (FFI) subscale scores and the total score from the baseline to week 2 and week 4, and patients’ self-reported level of improvement at week 4. Additional week 8 follow-up assessments with first-step pain VAS and FFI measurements will be arranged for the treatment group. Any adverse events will be recorded throughout the study to evaluate safety. An intention-to-treat approach will be used to analyze the study results.

**Discussion:**

This study will provide evidence on the efficacy and safety of electroacupuncture plus warm needling therapy as an alternative treatment method for heel pain. The findings will determine whether the treatment protocol is efficacious in relieving pain and improving foot function among older adults with heel pain. The study will also provide information for subsequent large-scale randomized controlled trials in the future.

**Trial registration:**

Chinese Clinical Trial Registry, ChiCTR1800014906. Registered on 12 February 2018.

**Electronic supplementary material:**

The online version of this article (10.1186/s13063-019-3572-4) contains supplementary material, which is available to authorized users.

## Background

Heel pain is a common foot disorder and most commonly involves pain surrounding the calcaneus inferiorly or posteriorly [1]. The typical complaint is pain and tenderness under the heel that is worse during weight-bearing, especially in the morning and when first moving after a period of inactivity, and is associated with tenderness over the calcaneal tuberosity at the commencement of weight-bearing activities. There are a variety of potential underlying causes, and treatment varies depending on the cause [1].

Heel pain is a common foot condition treated by health-care providers [2]. It is estimated to affect 10% of the general population at some time during life [3]. The frequency of the onset of disabling foot pain increases with age, and the prevalence of disabling foot pain will increase as the population ages [4]. The population-level prevalence of plantar heel pain in adults aged ≥ 50 years is 9.1% and for disabling plantar heel pain is 7.4% [5].

The risk of developing heel pain increases with increasing body mass index (BMI) and age [6]. Increasing BMI and age are associated with chronic plantar heel pain [7–9]. Chronic heel pain is most commonly reported by overweight, sedentary people aged 40–60 years and can occur throughout life [10, 11]. It has a significant negative impact on foot-specific and general health-related quality of life [11, 12].

Many treatment options are available, such as manual therapy, stretching exercises, taping, casted orthoses, night splints, extracorporeal shockwave therapy, education and counseling for weight loss, therapeutic exercise, dry needling, corticosteroid injection, local anesthetic injection, and surgery, with varying levels of evidence [2, 6, 13]. However, the best way to manage heel pain remains inconclusive. Heel pain may be a condition for which conservative management can be quite effective [14].

Previous systematic reviews have shown some positive evidence supporting the effectiveness of acupuncture in terms of providing significant benefits and improving pain and foot function related to heel pain, although the findings are inconclusive [15, 16]. Acupuncture treatments are widely variable with no consensus on which modality is most effective. In real-world practice, multiple treatments and rationales have been documented, but no single approach is dominant [17].

Studies have shown evidence for electroacupuncture analgesia [18]. Moxibustion has also been used for thousands of years to regulate meridians or channels and visceral organs of the human body. This is an integral therapeutic modality of acupuncture that uses ignited moxa floss to apply heat to certain points or areas of the body surface for treating diseases [19]. Moxibustion, which includes warm needling, is an effective treatment option in traditional Chinese medicine for acute and chronic pain [20, 21]. During warm needling, heat from the burning of moxa is transmitted to the corresponding acupuncture point by radiation as well as by conduction via the shaft of the needle, thereby stimulating deep tissue within the acupuncture point while warming the acupuncture point on the surface [22].

Studies have shown positive therapeutic effects of electroacupuncture [23], warm needling [24], or the combination of both [25] for heel pain, but with limitations in the study methodology. Combining these two modalities may provide an advantageous synergy in treating heel pain. Therefore, a well-designed randomized controlled trial is necessary to explore and determine the efficacy and safety of these methods in treating heel pain. Considering the significance of health problems associated with heel pain, a carefully designed randomized controlled clinical trial is proposed.

## Methods/design

### Study objective and hypothesis

This study aims to explore the efficacy and safety of electroacupuncture plus warm needling therapy for treating heel pain among older adults in Hong Kong. The study will examine whether electroacupuncture plus warm needling therapy can reduce heel pain intensity and improve foot function for patients with heel pain. We hypothesized that electroacupuncture plus warm needling therapy is better than the waitlist control in reducing pain intensity and improving foot function for patients with heel pain. This hypothesis is based on the superiority of electroacupuncture plus warm needling therapy over the waitlist control.

### Study design and setting

This study is a prospective, nonblinded, parallel-group, randomized controlled trial with a 1:1 ratio of patients allocated to receive electroacupuncture plus warm needling therapy (i.e., treatment group) or those allocated to the waitlist control (i.e., control group, who will receive delayed active acupuncture treatment after a week 4 assessment). The trial will be conducted at Pok Oi Hospital—The Chinese University of Hong Kong Chinese Medicine Centre for Training and Research (Shatin). The study protocol was developed based on the Standard Protocol Items: Recommendations for Interventional Trials (SPIRIT) [26]. The SPIRIT 2013 checklist is presented in Additional file 1. The study design and reporting of the study will follow the recommendations of the Consolidated Standards of Reporting Trials (CONSORT) [27], the STandards for Reporting Interventions in Clinical Trials of Acupuncture (STRICTA) [28], and the STandards for Reporting Interventions in Clinical Trials of Moxibustion (STRICTOM) [29].

### Study population

This study will focus on individuals with heel pain who are 50–80 years old. The eligibility criteria were developed primarily to enroll the appropriate individuals and to exclude individuals with serious diseases or contraindications to the treatments.

### Inclusion criteria

Individuals will be recruited for the study if they satisfy all of the following criteria:

1. Aged 50–80 years
2. Heel pain that is characterized by worsening when standing or when walking after getting up in the morning; the pain is relieved after walking for a while and worsens after a long period of walking [30]
3. Heel pain with marked tenderness at the plantar aspect and lateral aspect of the calcaneal tuberosity [30]
4. Heel pain that is unilateral or bilateral
5. Pain that is acute or chronic, with the degree of heel pain experienced on taking the first few steps rating ≥ 50 mm on a visual analog scale (VAS) of 0–100 mm at the time of recruitment
6. Agree to sign the informed consent form

### Exclusion criteria

Individuals will be excluded if they possess any of the following criteria:

1. Pain in another area that is more severe than the heel pain
2. Loss of plantar foot sensation
3. Open wounds, tumors, or skin ulceration on the painful heel (or heels) and surrounding area
4. Documentation of a fracture or abnormalities at the painful heel (or heels) within 4 weeks that are unsuitable for the study treatments
5. Acupuncture and/or moxibustion treatment for the same heel pain during the previous month
6. Expected ongoing co-interventions (i.e., medication or alternative treatments or both) during the study period
7. Previous surgery to the painful heel (or heels) or are scheduled to have surgery during the study period
8. Severe needle phobia
9. Known hypersensitive reaction after acupuncture and moxibustion treatment or an inability to cooperate with the acupuncture and moxibustion procedure
10. Diagnosis of cancer of any nature within 5 years
11. Known severe disease of the heart, brain, lung, liver, kidney, or hematopoietic system
12. A cardiac pacemaker
13. Pregnancy, are breastfeeding, or are expecting to become pregnant during the study period (a pregnancy test will be performed for child-bearing women whose last menstrual period was beyond 28 days and who are not in menopause or have not undergone sterilization)
14. Known severe psychiatric or psychological disorder
15. Other factors that have been deemed unsuitable for participation, as assessed by the investigators
16. Engagement in any other clinical trial during the study period
17. Pending foot-related litigation or disability claims
18. Lack of capability to understand and answer the questions posed by the assessors in the study

### Recruitment

**Fig. 1:**
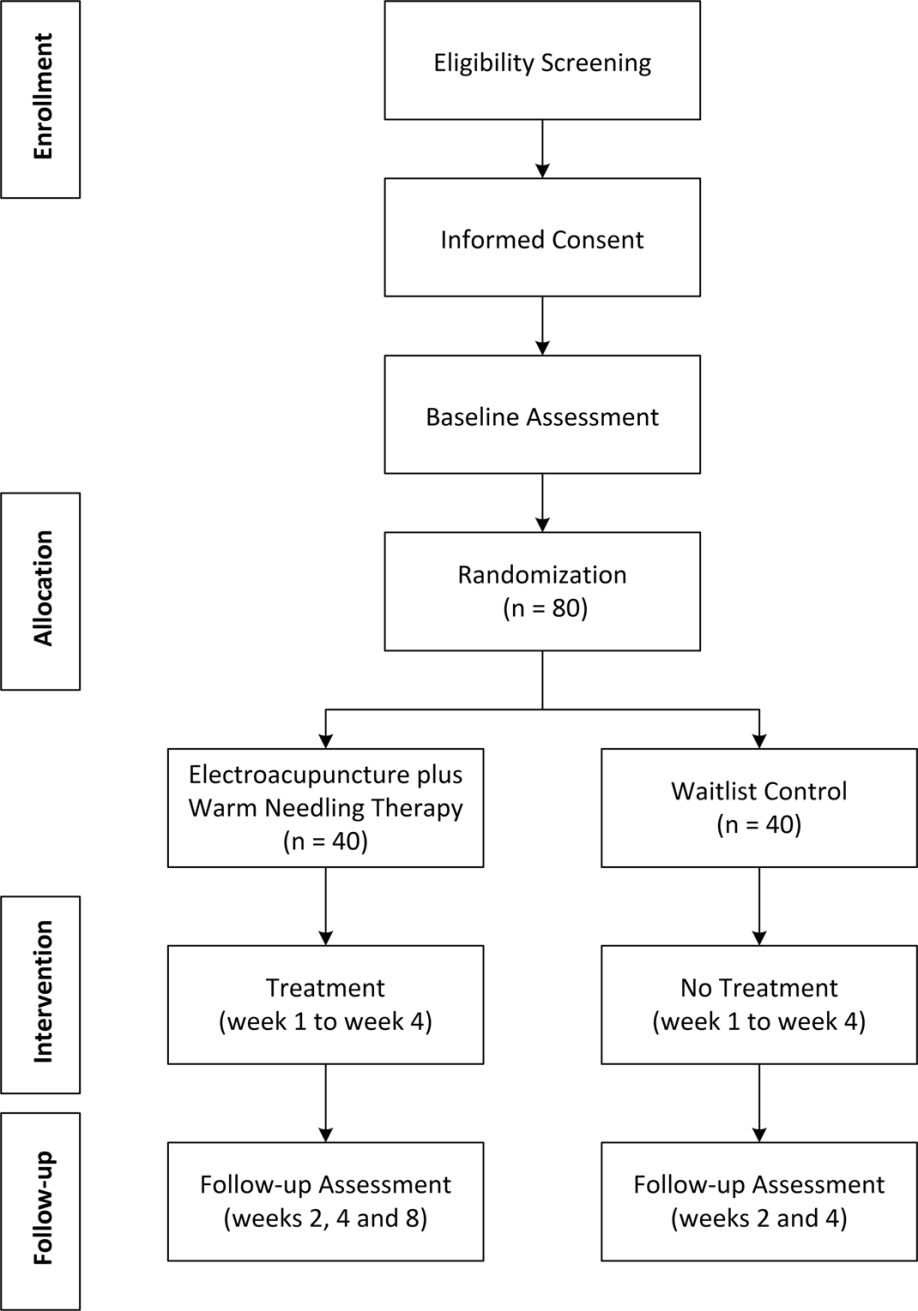
Flowchart of study procedures

**Fig. 2:**
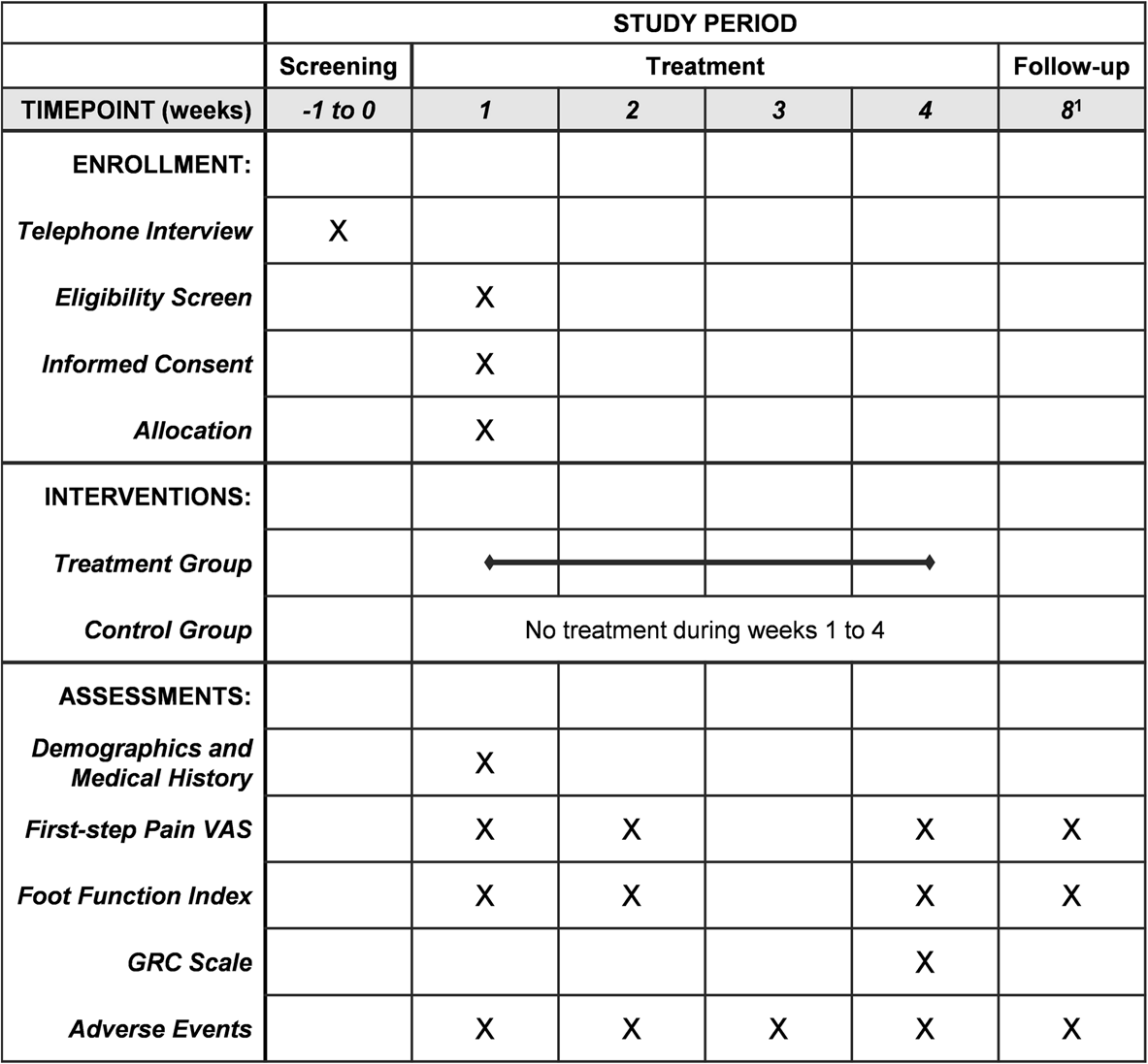
Schedule of enrollment, interventions, and assessments. ^1^Only the treatment group will undergo the week 8 assessment and visits for ± 4 days are allowed. VAS visual analog scale, GRC Global Rating of Change

Individuals will be recruited through posters on bulletin boards at all Chinese medicine centers, polyclinics, and mobile clinics under the management of Pok Oi Hospital, through social media, and by cross-referral from the Chinese medicine practitioners of these clinics. Individuals interested in participating in the study will be referred to or will self-approach Pok Oi Hospital—The Chinese University of Hong Kong Chinese Medicine Centre for Training and Research (Shatin) to undergo eligibility assessment. They will be prescreened through a telephone interview. Potential candidates for recruitment to the study will have a face-to-face interview arranged to confirm their eligibility. During the interview, investigators will explain the overall objectives and nature of the study, describe the informed consent process, and assess the candidates’ eligibility. After an individual voluntarily reviews and signs the informed consent form, information such as sex, age, height, weight, BMI, and medical history will be collected. In addition, baseline assessment of primary and secondary outcome measures will be administered. An independent staff person who is not involved in the study will thereafter randomly allocate the study participants to one of two groups. A schedule for the treatment procedure will be assigned. The study flow is shown in Fig. 1. Figure 2 presents the schedule of enrollment, interventions, and assessments.

### Follow-up visits

The acupuncture and moxibustion used in this study are based on traditional Chinese medicine theory and a review of the literature. A previous study [31] has shown that warm needling acupuncture has a synergistic and more effective analgesic effect than acupuncture without heat or than heat applied to the skin surface. The treatment group will undergo a total of six treatment sessions because previous articles and reviews have shown that six or more acupuncture treatments were significantly associated with positive outcomes [32, 33]. The treatment regimen in this study will involve six treatment sessions of electroacupuncture plus warm needling therapy within a 4-week period on a regular basis (i.e., one treatment session every 3–5 days). The treatments will be provided at no cost to the participants. Assessments and a questionnaire will be administered on the day of the treatment session at week 2 (i.e., after the third treatment), at week 4 (i.e., after the sixth treatment), and at week 8 (for longer-term treatment effect evaluation).

No intervention will be provided for the waitlist control group during the study period. The participants will be informed that they are required to visit the Chinese Medicine Centre at week 2 and week 4 for study assessments. Considering that heel pain is an annoying condition and may affect a patient’s daily life, it may not be appropriate to start treatment after a longer waiting period. Therefore, participants will be scheduled to receive six acupuncture treatments for heel pain for free at their own discretion after the week 4 study assessments. No week 8 study assessments will be arranged for the control group because that would not provide a valid comparison with the treatment group.

To promote subject retention and complete follow-up, the study investigators will make every reasonable effort to follow the participants for the entire study period and provide timely reminders to the patients regarding their treatment or assessment schedule. A designated telephone line will be provided to all study participants to report adverse events during office hours.

### Randomization and blinding

The treatment allocation into the electroacupuncture plus warm needling therapy or waitlist control groups of each eligible individual in the study will be determined by a randomization process. The random allocation to the two arms of the study will be achieved using Random Allocation Software [34] following a balanced 1:1 pattern (electroacupuncture plus warm needling therapy:waitlist control) with a block size of 10. This randomization process will be conducted by an independent staff person who is not involved in the study to guarantee concealment of the allocations. The information on the allocation list will remain strictly confidential. The allocations will be concealed and sequentially numbered; opaque, sealed envelopes will be used to contain the randomization assignments.

### Background of the practitioners

The study treatments will be performed by Registered Chinese Medicine Practitioners (RCMPs) in Hong Kong who each have more than 3 years of experience in acupuncture and are trained in administering warm needling acupuncture. The RCMPs in this study should strictly adhere to the study protocol and train to be familiar with administering the study treatments. The practitioners will be asked to maintain the same attitude toward each individual to avoid any psychological influences.

### Treatment procedure

During each treatment session, the treatment group will receive electroacupuncture plus warm needling therapy on the affected heel(s) at one Ashi point [19, 35] and at Shuiquan (KI5, the cleft point of the Kidney Meridian) [36, 37]. The Ashi point is a tender point that is not included in the classic acupuncture points [19, 36]; it is frequently used in treating heel pain with an effect on relieving pain [15, 24, 25]. The literature has shown that combining one or more cleft point in acupuncture treatment for heel pain has a more effective result [38]. The location of the Ashi point used in this study is the anteromedial aspect of the painful heel (or heels) with marked tenderness, and KI5 is determined based on the guidelines presented in the World Health Organization Standard Acupuncture Point Locations in the Western Pacific Region [37]. For individuals with pain in both heels, treatment will be provided bilaterally.

Treatments will be administered using sterile, single-use, disposable acupuncture needles with guide tubes (size, 0.25 mm × 40 mm; Wuxi Jiajian Medical Instrument Co., Ltd, Wuxi, China). During the treatment, the room temperature will be maintained at 25 ± 3 °C, and the individual will lie in the supine position with the affected heel(s) exposed. The skin will first be sterilized before needle insertion. After that, needles will be inserted perpendicularly for all selected acupuncture points to a depth of 20 mm, followed by the stimulation methods of lifting and thrusting combined with twirling and rotating the needles evenly to achieve a dull ache, numbness or heaviness sensation, called *deqi* [39, 40]. All needles will be retained in situ for 30 min. During the retaining period, electrical stimulation and warm needling therapy will be administered simultaneously.

Paired electrodes of the electroacupuncture apparatus (Hwato SDZ-III; Suzhou Medical Appliance Factory, Suzhou, China) will be connected to the needles at the Ashi point and KI5 on the same side of the heel. Electrical stimulation will be delivered with a dense-disperse wave and a frequency of 2 Hz. The intensity of the electric current will be increased to the patient’s maximum tolerance and then slightly reduced to a bearable and comfortable level.

The bottom of a cylinder-shaped moxa stick (Changsha Furong Huai, Changsha, China), which measures 12 mm in diameter and 15 mm in length and weighs 0.7 ± 0.05 g, will be ignited and attached to the needle handle for each acupuncture point. The needle handle will be inserted into the center of the cylinder-shaped moxa stick. The ignited end will be oriented toward the skin and placed 3 cm from the skin surface, which is an appropriate and safe distance for administering moxibustion [41–43]. When the first moxa stick has burned fully, the ashes will be removed, the second stick will be placed, and the same procedure will be followed. In total, two moxa sticks will be applied to each acupuncture point. The burning time for each moxa stick is approximately 10 min. The total moxibustion time for two moxa sticks will be approximately 20 min, an advisable time and within the effective thermal stimulation range for moxibustion [41, 44]. The individual will feel a sense of local warmth without burning pain, and the surrounding skin will become mildly red during the procedure. The needles will be removed after the retention time and at the end of moxibustion.

Precautionary measures should be taken for warm needling therapy. In the process of inserting the ignited moxa stick into the needle handle, the practitioner holds the junction of the needle body with one hand while inserting the moxa stick into the needle at the inserted acupuncture points gently with the other hand to prevent skin burns. After attaching the moxa stick to the needle handle, the practitioner should ensure that the stick is fully fixed into the needle handle to prevent it from falling onto the skin surface. When placing the second moxa stick and removing the needles at the end of the treatment, the practitioner should take note of the temperature of the needles to prevent burns. The practitioner must be aware of the individual’s sensitivity to temperature and pain, and advise the individual not to let the acupuncture points become uncomfortably hot. The individual will be closely monitored during the entire procedure to ensure safety and to guarantee that any accident is handled in a timely manner [45].

### Permitted and prohibited concomitant treatments

During the study period, which includes the follow-up period, individuals will not be allowed to use other pain control interventions in relation to their heel pain, which includes but is not limited to other acupuncture treatments, bone setting, physiotherapy, and medication. Such interventions could influence the study results. The practitioner will advise the participants to avoid other treatments, and this advice will be reinforced by investigators during the entry period and at each evaluation time point. In the event that an individual has utilized other cointerventions for heel pain, such as taking pain-relief medications, this information should be reported to investigators during each evaluation period, and the information will be recorded for analysis. However, general light exercises and self-massage will be allowed for all individuals participating in the study.

### Study termination

The study will be stopped if the principal investigator believes that there is an unacceptable risk of serious adverse events during the study treatments.

### Outcome measurements

In the study, the unit of analysis will be selected on an individual basis, regardless of whether one or both heels were affected [46]. Individuals with pain in one heel or in both heels will be included without discrimination. Outcomes for heel pain and foot function will be assessed in heels with more severe symptoms.

#### Primary outcome measure

The primary outcome measure will be the mean change in the pain intensity of the first step (i.e., first-step pain) between the baseline and after treatment (i.e., week 4). First-step pain is the pain experienced after a long period of not bearing weight, such as upon first stepping out of bed in the morning, and is a common complaint of individuals with plantar heel pain [3, 15], measured with a 100-mm VAS. The VAS for pain is a widely used measurement of pain intensity [47–49]. It is a continuous scale ranging from 0 to 100, where 0 represents “no pain” and 100 represents the “worst imaginable pain”.

#### Secondary outcome measures

Secondary outcome measures include the mean change in the first-step pain VAS score from the baseline to week 2. The same VAS used for the primary outcome measure will be applied again. In addition, the mean change in the Foot Function Index (FFI) subscale scores and total score from the baseline to week 2 and week 4 and the patients’ self-reported level of improvement at week 4 will also be measured as secondary outcomes.

The FFI is a frequently-used self-reporting measure of foot-specific pain and disability; it has good reliability and validity [49–51]. In this study, the Chinese version of the validated FFI [52] will be used. It consists of 23 items divided into three subscales: Pain (nine items), Disability (nine items), and Activity Limitation (five items). Each item will be rated on a VAS, which is a horizontal line without numbers or divisions. The poles are labeled “no pain” and “worst pain imaginable” for the Pain subscale; “no difficulty” and “so difficult unable” for the Disability subscale; and “none of the time” and “all of the time” for the Activity Limitation subscale. Each item score is derived by dividing the attached horizontal line into 10 equal segments and assigning a number ranging from 0 to 9 to each segment. A subscale score is calculated by summing the item scores for the subscale, dividing it by the maximum total possible score for all of the subscale items that the individual indicated as applicable, and then multiplying the value by 100. Any item marked “not applicable” will be excluded from the total possible score. Subscale scores range from 0 to 100. The total foot function score is derived by averaging the three subscale scores together. A higher score indicates greater impairment and a lower level of functioning (i.e., greater disability or loss of function).

The participants’ self-reported level of improvement will be measured on the 15-point Global Rating of Change (GRC) scale [53, 54]. Each number on the 15-point GRC scale represents a different change in health status. The numbers range from − 7 to + 7 as follows: − 7, “a very great deal worse”; − 6, “a great deal worse”; − 5, “a good deal worse”; − 4, “moderately worse”; − 3, “somewhat worse”; − 2, “a little worse”; − 1, “almost the same, hardly any worse at all”; 0, “no change”; + 1, “almost the same, hardly any better at all”; + 2, “a little better”; + 3, “somewhat better”; + 4, “moderately better”; + 5, “a good deal better”; + 6, “a great deal better”; and + 7, “a very great deal better”.

#### Subsidiary observation

For the longer-term evaluation of the effects of electroacupuncture plus warm needling therapy in the treatment group, a subsidiary assessment will be conducted at week 8 for the primary and secondary outcome measures.

#### Adverse events

For safety concerns, all unexpected and unintended responses that are not necessarily related to the study treatments will be recorded with a detailed explanation such as the time of occurrence, duration of symptoms, seriousness of symptoms (mild, moderate, or severe [55]), management measures, time of adverse reaction disappearance, and causality categorization (i.e., “certain”, “probable or likely”, “possible”, “unlikely”, “unclassified”, or “unclassifiable” [56]) at every visit on an adverse event report. Adverse events that are associated with acupuncture and moxibustion include acute pain, bruising, bleeding, burns, blisters, dizziness, anxiety, and infection [55, 57]. Investigators will record and manage all adverse events, regardless of whether it is relevant to the study treatment.

#### Dropouts and withdrawals

All dropouts and attrition during the course of the study will be monitored, and the respective reasons for withdrawal will be recorded.

### Data management

Data will be collected after acquiring the signed consent forms from the participants. All data from the visits will be recorded in the medical records, and all data from the outcome assessments will be collected using paper-based questionnaires. The collected data will be anonymized and recorded on case report forms. All data will be confidential, and access to the data will be limited to delegated research personnel. All study-related physical data and information will be stored securely in a double-locked cabinet in a dedicated office at the study site that is secured by a code-operated lock and is only accessible by delegated research personnel. All electronic datasets will be password protected. The participants’ study information will not be released outside the study without obtaining the written permission of the participant. After the trial, all documents will be preserved in the secure research archives at the study site for 7 years and will be deleted after the completion of the storage period.

### Statistical analysis

#### Sample size calculation

The sample size calculation will be based on the primary outcome and evaluated with the OpenEpi statistical program [58]. The sample size estimate is based on a two-arm normal design. A two-sided test with an α level of 0.05 and 80% power detects a minimally important difference of 19 mm with a standard deviation of 28 mm (first-step pain as measured by the VAS) [54]. With an assumed attrition rate of 10%, each group will require 40 individuals, which implies a total sample size of 80 individuals for the entire trial.

#### Statistical methods and analysis

The statistical analysis will be conducted based on the intention-to-treat principle. Continuous variables will be summarized using the means and standard deviations or the means and 95% confidence intervals. Categorical variables will be summarized using counts and percentages.

The measurement data will first be tested using a normality test. For the descriptive analysis, data will be evaluated for normality and log-transformed when necessary. Two-sample *t* tests or the Wilcoxon rank-sum tests for continuous data and Pearson chi-squared tests or Fisher’s exact tests for categorical data will be conducted, depending on whether the data are normally distributed or skewed.

For the primary outcome, the paired *t* test or Wilcoxon signed-rank test will be used to perform a within-group comparison on the mean change between scores assessed before and after treatment. The two-sample *t* test or Wilcoxon rank-sum test will be used for comparisons between groups. The mean differences in other secondary outcome measures will be analyzed by following the same methodology used in the primary outcome measurement. In addition, longitudinal mixed-effects model repeated-measures tests will also be conducted to analyze the mean change in outcome measurements from the baseline to the end of the trial between the two study groups.

Analyses of data in this study will be performed using SPSS (IBM Corp, Armonk, NY, USA) and/or Stata software (StataCorp LLC, College Station, TX, USA). Multiple imputation techniques will be used to manage missing values. All tests will be two-sided, and *P* < 0.05 will be considered statistically significant.

### Study monitoring

A dedicated team from the Hospital Authority Chinese Medicine Department (HACMD) that is completely uninvolved in the running of the study and has no competing interests will be responsible for monitoring the study progress. Pre-trial and post-trial onsite inspections and quarterly monitoring meetings will be held to oversee the progress of the study and ensure it is conducted, recorded, and reported in accordance with the protocol, Good Clinical Practice guidelines, and applicable regulatory requirements. Although a separate data monitoring committee will not be established, the HACMD will serve this function to ensure scientific validity, scientific integrity, and data accuracy through the regular monitoring of this clinical trial.

### Ethical considerations and dissemination of information

The study will be conducted in compliance with local laws. It will adhere to the common guidelines of the Declaration of Helsinki for medical research involving humans [59, 60] and the International Council for Harmonisation of Technical Requirements for Pharmaceuticals for Human Use–Good Clinical Practice (ICH-GCP) [61]. Ethical approval was obtained before the commencement of the study. The ethical validity of the study was approved by the Joint Chinese University of Hong Kong–New Territories East Cluster Clinical Research Ethics Review Committee (CREC Ref. No. 2017.600-T). Any modifications to the protocol are reported to this committee, and amendment approval should be obtained before any changes can take place. The study has been registered in the Chinese Clinical Trials Registry (ChiCTR1800014906). All individuals will receive sufficient information about the study, and written informed consent will be obtained from each person before enrollment. We plan to publish the results of this study in peer-reviewed journals or academic conference proceedings.

## Discussion

Patients who experience heel pain often suffer from pain and functional limitations. The prevalence of disabling foot pain will increase as the population ages. Acupuncture and moxibustion have been used to treat pain and functional limitations of heel pain for many years with positive results. Several previous studies have shown the positive therapeutic effects of electroacupuncture [23], warm needling [24], or the combination of both [25] for heel pain. According to these studies, single or combination therapy decreased pain intensity and symptoms of pain in patients with heel pain. However, because of the limitations of the study methodologies, little scientific evidence exists supporting the effectiveness of the methods. Therefore, we designed our study with an adequate methodology that follows international guidelines and uses validated assessment tools.

The objective of treating heel pain is to improve the quality of life by providing pain relief and preserving a patient’s foot function. The present study is an open-label, two-armed, parallel-group, randomized controlled trial that is designed to investigate the efficacy and safety of the combination therapy of electroacupuncture plus warm needling for treating patients with heel pain.

One limitation of the study is the possibility of a high risk of bias with regard to blinding because we used a waiting list as the control instead of a sham procedure. Owing to the nature of the acupuncture and moxibustion interventions, neither the study participants nor the acupuncturists can be blinded to the allocation and treatment stages.

This rigorously designed evidence-based clinical trial will evaluate whether electroacupuncture plus warm needling therapy is efficacious and safe in relieving pain and improving foot function among older adults with heel pain in Hong Kong. The present protocol may demonstrate electroacupuncture plus warm needling therapy as an effective and safe treatment option for patients with heel pain. The results obtained in the proposed study will provide valuable information and a solid foundation for future larger-scale clinical studies on the same topic. The study will provide evidence to determine whether the treatment protocol, which involves the combination of acupuncture and moxibustion, could be a potentially effective treatment alternative for heel pain.

## Trial status

The study commenced after receiving ethical approval. Participant recruitment began on 1 May 2018 and is expected to be completed on before 31 August 2019. This is version 4 of the protocol, dated 19 September 2018.

## Additional file

Additional file 1: Standard Protocol Items: Recommendations for Interventional Trials (SPIRIT) 2013 checklist: recommended items to address in a clinical trial protocol and related documents (DOC 125 kb)

## Data Availability

The datasets used and/or analyzed during the current study will be available from the corresponding author after the completion of the study upon reasonable request.

## Abbreviations

BMI: Body mass index
CONSORT: Consolidated Standards of Reporting Trials
FFI: Foot Function Index
GRC: Global Rating of Change
HACMD: Hospital Authority Chinese Medicine Department
ICH-GCP: International Council for Harmonisation of Technical Requirements for Pharmaceuticals for Human Use–Good Clinical Practice
RCMP: Registered Chinese Medicine Practitioner
SPIRIT: Standard Protocol Items: Recommendations for Interventional Trials
STRICTA: STandards for Reporting Interventions in Clinical Trials of Acupuncture
STRICTOM: STandards for Reporting Interventions in Clinical Trials of Moxibustion
VAS: Visual analog scale
